# Supplementary figures and images for: Composite core set construction and diversity analysis of Iranian walnut germplasm using molecular markers and phenotypic traits
Source: PLoS One. 2021 Mar 16;16(3):e0248623. doi: 10.1371/journal.pone.0248623 (PMC7963058; doi:10.1371/journal.pone.0248623)

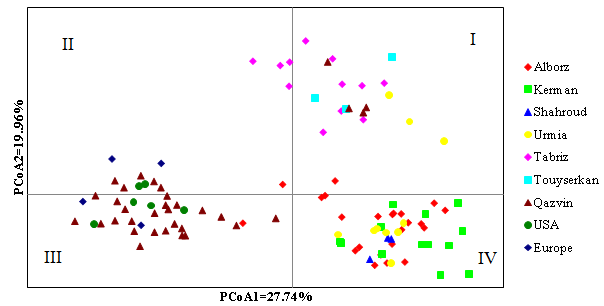


**S1 Fig.** Principal coordinate analysis of the accessions based on AFLP markers

Supplement: S1 Fig — (DOCX) [file pone.0248623.s002.docx]
