# Supplementary material for: Composite core set construction and diversity analysis of Iranian walnut germplasm using molecular markers and phenotypic traits
Source: PLoS One. 2021 Mar 16;16(3):e0248623. doi: 10.1371/journal.pone.0248623 (PMC7963058; doi:10.1371/journal.pone.0248623)
Supplement: S2 Fig — Each individual is shown by a vertical line with one to three colored segments, according to its estimated membership probabilities (Q). (DOCX) [file pone.0248623.s003.docx]

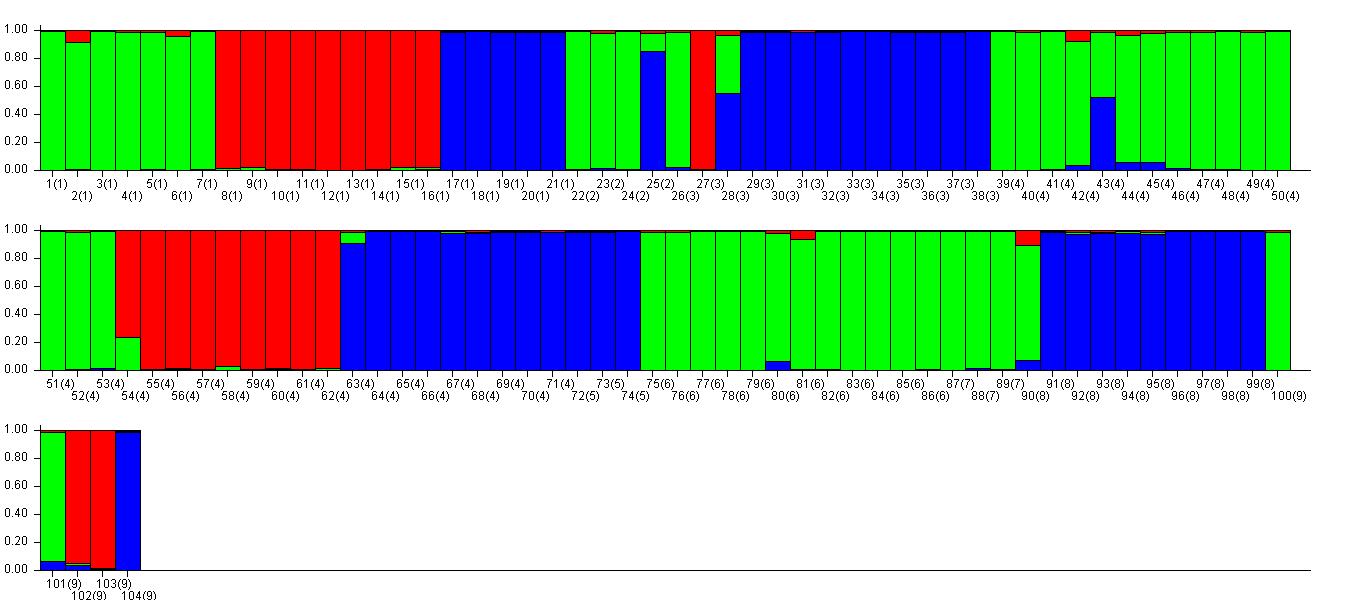


**S2 Fig.** Pattern of individual assignments into three subsets (K = 3) using the STRUCTURE software. Each individual is shown by a vertical line with one to three colored segments, according to its estimated membership probabilities (Q)
