# Supplementary material for: Composite core set construction and diversity analysis of Iranian walnut germplasm using molecular markers and phenotypic traits
Source: PLoS One. 2021 Mar 16;16(3):e0248623. doi: 10.1371/journal.pone.0248623 (PMC7963058; doi:10.1371/journal.pone.0248623)
Supplement: S1 Table — (DOCX) [file pone.0248623.s004.docx]

|  | **S1 Table.** Descriptors for the qualitative traits utilized | | | | | | | | | |
| --- | --- | --- | --- | --- | --- | --- | --- | --- | --- | --- |
| **Trait** |  | **Code and state** | |  |  |  |  |  |  |  |
|  | **Abbr.** | **1** | **2** | **3** | **4** | **5** | **6** | **7** | **8** | **9** |
| **Nut Size** | NS | very small | * | small | * | medium | * | large |  | very large |
| **Shape in lon­gitudinal section through suture** | SLSTS | circular | triangular | broad ovate | ovate | broad trapezium | trapezium | broad elliptic | elliptic | * |
| **Shape in longitudinal section perpendicular to suture** | SLSPS | circular | triangular | broad ovate | ovate | broad trapezium | trapezium | broad elliptic | elliptic | cordate |
| **Shape in cross section** | SCS | oblate | circular | elliptic | * | * | * | * | * | * |
| **Shape of base perpendicular to suture** | SPS | cuneate | rounded | truncate | emarginate | * | * | * | * | * |
| **Shape of apex perpendicular to suture** | SAPS | pointed | rounded | truncate | emarginate | * | * | * | * | * |
| **Prominence of apical tip** | PAT | * | * | weak | * | medium | * | strong | * | * |
| **Position of pad on suture** | PoPS | on upper half | on upper 2/3 | on whole length | * | * | * | * | * | * |
| **Prominence of pad on suture** | PrPS | * | * | weak | * | medium | * | strong | * | * |
| **Width of pad on suture** | WPS | * | * | narrow | * | medium |  | broad | * | * |
| **Depth of grove along pad on suture** | DGAPS | * | * | shallow | * | medium |  | deep | * | * |
| **Structure of surface of shell** | SSS | slightly grooved | moderately grooved | strongly grooved | embossed | * | * | * | * | * |
| **Adherence of two halves of shell** | ATHS | very weak | * | weak | * | medium | * | strong | * | very strong |
| **Thickness of dividing membranes** | TDM |  | * | thin | * | medium | * | thick | * | * |
| **Ease of removal** | ER | very easy | * | easy | * | medium | * | difficult | * | * |
| **Intensity of ground color** | IGC | very light | * | light | * | medium | * | dark | * | * |
| **Kernel Size** | KS | very small | * | small | * | medium | * | large | * | very large |

**NS**: Nut Size, **SLSTS**: Shape in lon­gitudinal section through suture, **SLSPS**: Shape in longitudinal section perpendicular to suture, **SCS**: shape in cross section, **SPS**: Shape of base perpendicular to suture, **SAPS**: Shape of apex perpendicular to suture, **PAT**: Prominence of apical tip, **PoPS**: Position of pad on suture, **PrPS**: Prominence of pad on suture, **WPS**: Width of pad on suture, **DGAPS**: Depth of grove along pad on suture, **SSS**: Structure of surface of shell, **ATHS**: Adherence of two halves of shell, **TDM**: Thickness of dividing membranes, **ER**: Ease of removal, **IGC**: Intensity of ground color, **KS**: Kernel Size.
