# Supplementary material for: Composite core set construction and diversity analysis of Iranian walnut germplasm using molecular markers and phenotypic traits
Source: PLoS One. 2021 Mar 16;16(3):e0248623. doi: 10.1371/journal.pone.0248623 (PMC7963058; doi:10.1371/journal.pone.0248623)
Supplement: S2 Table — (DOCX) [file pone.0248623.s005.docx]

**S2 Table.** Sequences of oligonucleotide adaptors and primers used for AFLP

| Adaptor/primer | Sequence |
| --- | --- |
| Adaptors | |
| *Eco*RI adaptors | 5´-CTC GTA GAC TGC GTA CC-3´ |
|  | 3´-CAT CTG ACG CAT GGT TAA-5´ |
| *Mse*I adaptors | 5´-GAC GAT GAG TCC TGA G-3´ |
|  | 3´-TAC TCA GGA CTC AT-5´ |
| Pre selective amplification |  |
| M000 | GAC TGC GTA CCA AAT |
| E000 | GAT GAG TCC TGA GTA |
| Selective amplification |  |
| M-CAT | GAT GAG TCC TGA GTA ACA T |
| M-GAG | GAT GAG TCC TGA GTA AGA G |
| M-CAG | GAT GAG TCC TGA GTA ACA G |
| E-CT | GAC TGC GTA CCA ATT CCT |
| E-GT | GAC TGC GTA CCA ATT CGT |
| E-AT | GAC TGC GTA CCA ATT CAT |
