# Supplementary material for: Composite core set construction and diversity analysis of Iranian walnut germplasm using molecular markers and phenotypic traits
Source: PLoS One. 2021 Mar 16;16(3):e0248623. doi: 10.1371/journal.pone.0248623 (PMC7963058; doi:10.1371/journal.pone.0248623)
Supplement: S3 Table — (DOCX) [file pone.0248623.s006.docx]

**S3 Table.** Internal and external similarity measures of groups and membership of walnut in each cluster corresponding to Fig 1

| **Esdev** | **Esim** | **Isdev** | **Isim** | **Size** | **Cluster** |
| --- | --- | --- | --- | --- | --- |
| 0.047 | 0.079 | 0.061 | **0.580** | 40 | 1 |
| 0.062 | 0.047 | 0.050 | 0.412 | 12 | 2 |
| 0.050 | 0.096 | 0.076 | 0.444 | 10 | 3 |
| 0.065 | 0.081 | 0.076 | 0.389 | 16 | 4 |
| 0.071 | 0.128 | 0.064 | 0.425 | 12 | 5 |
| 0.062 | **0.010** | .060 | 0.403 | 14 | 6 |

**Isim**: internal similarity, **Isdev**: internal standard deviation, **Esim**: external similarity, **Esdev**: external standard deviation
