# Supplementary material for: Composite core set construction and diversity analysis of Iranian walnut germplasm using molecular markers and phenotypic traits
Source: PLoS One. 2021 Mar 16;16(3):e0248623. doi: 10.1371/journal.pone.0248623 (PMC7963058; doi:10.1371/journal.pone.0248623)
Supplement: S4 Table — (DOCX) [file pone.0248623.s007.docx]

**S4 Table.** Analysis of molecular variance (AMOVA) on based on AFLP markers of 104 accessions

| **%**  **Variance** | **Variance Components** | **Sum of Squares** | **Source of variance** |
| --- | --- | --- | --- |
| 6.32 | 1.856 | 377.28 | Between populations |
| 93.98 | 27.497 | 2612.26 | Within populations |
| ------- | 29.35 | 2989.53 | Total |
