# Supplementary material for: Composite core set construction and diversity analysis of Iranian walnut germplasm using molecular markers and phenotypic traits
Source: PLoS One. 2021 Mar 16;16(3):e0248623. doi: 10.1371/journal.pone.0248623 (PMC7963058; doi:10.1371/journal.pone.0248623)
Supplement: S5 Table — (DOCX) [file pone.0248623.s008.docx]

**S5 Table.** Range median, mean, coefficient of variation and Shannon Diversity Index for the traits evaluated

| **Traits** | **Unit** | **Min.** | **Max.** | **Median** | **Mean** | **CV** | **H**´ | **Number of states** |
| --- | --- | --- | --- | --- | --- | --- | --- | --- |
| NS | Code | 3 | 9 | 5 | 5.14 | 26.54 | 0.641 | 4 |
| SLSTS | Code | 1 | 8 | 4 | 4.05 | 57.17 | 0.934 | 8 |
| SLSPS | Code | 1 | 9 | 5 | 4.34 | 52.42 | 0.920 | 9 |
| SCS | Code | 1 | 3 | 2 | 1.61 | 36.29 | 0.771 | 3 |
| SPS | Code | 1 | 3 | 2 | 2.32 | 28.66 | 0.729 | 3 |
| SAPS | Code | 1 | 4 | 2 | 2.33 | 35.60 | 0.798 | 3 |
| PAT | Code | 1 | 3 | 2 | 1.62 | 38.02 | 0.811 | 3 |
| PoPS | Code | 2 | 3 | 2 | 2.17 | 17.40 | 0.399 | 3 |
| PrPS | Code | 3 | 7 | 5 | 4.86 | 24.30 | 0.799 | 3 |
| WPS | Code | 3 | 7 | 5 | 5.32 | 18.29 | 0.613 | 3 |
| DGAPS | Code | 3 | 7 | 5 | 4.72 | 26.27 | 0.840 | 3 |
| SSS | Code | 1 | 4 | 3 | 2.87 | 15.31 | 0.373 | 4 |
| ATHS | Code | 1 | 9 | 5 | 5.26 | 33.02 | 0.799 | 5 |
| TDM | Code | 1 | 5 | 3 | 3.44 | 25.58 | 0.468 | 3 |
| ER | Code | 1 | 9 | 3 | 3.04 | 41.59 | 0.562 | 4 |
| IGC | Code | 1 | 7 | 5 | 4.60 | 34.76 | 0.823 | 4 |
| KS | Code | 1 | 9 | 5 | 4.66 | 34.54 | 0.756 | 5 |

CV**:** Coefficient of Variation (SD/mean *100), **H**´**:** Shannon diversity index. **Number of states**: number of classes for each trait
