# Supplementary material for: Composite core set construction and diversity analysis of Iranian walnut germplasm using molecular markers and phenotypic traits
Source: PLoS One. 2021 Mar 16;16(3):e0248623. doi: 10.1371/journal.pone.0248623 (PMC7963058; doi:10.1371/journal.pone.0248623)
Supplement: Attachment — Submitted filename: Resonse to revewiers 17-01-21.docx [file pone.0248623.s010.docx]

***Dear Ji-Zhong***

Thank you and the kind reviewer very much for the valuable comments. We made the proposed corrections, except in cases that there were some clarifications could be found here in “Response to reviewer”:

Thank you very much again and best wishes,

*Darab Hassani*

*Corresponding author*

Comment:

Line 19-20: Not only qualitative traits was used. Thus, I suggest to mention also the 18 quantitative traits. " the phenotypic variability of 17 and 18 qualitative and quantitative traits, respectively. See information on line 246-247 ("To determine a core collection, initial selections were made based on use of AFLP (CC1), quantitative phenotypic traits (CC2), and qualitative phenotypic data (CC3)."

*Response:*

*Correction has been done.*

Comment:

Please, use the same term throughout the manuscript, i.e. traits. Some sentences use qualitative traits while in others use qualitative characteristics.

*Response:*

*Correction has been done.*

Comment:

Line 99: According to table S2, 9 selective AFLP primer combinations were used. (M-CAT; M-GAG; M-CAG) x (E-CT; E-GT; E-AT) which gives 3X3= 9. Why 13 are mentioned in the manuscript?

*Response:*

*The number of primers initially were 13. So, for determining the genetic variability among the walnut genotypes, at the beginning 13 AFLP primers were evaluated. Among the evaluated primers, five primer combinations showed polymorphism with a 499 total and 197 polymorphic fragments.” It is mentioned in line197-198*

Comment:

Line 104-105: The sentence "The AFLP primer combinations (MseI, EcoRI) were labeled with infrared dyes IRD-700 andIRD-800 at the 5´ end, accompanied by two and three selective nucleotides at the 3´end." should be corrected according to the Table S2, in which MseI has three selective nucleotides while EcoRI two. Thus the sentence should be rewriten as:...accompanied by three and two selective nucleotides at the 3´end, respectively".

*Response:*

*Correction has been done.*

Comment:

Line 106: The unit microliters should be corrected: "5 µ of extracted DNA at a concentration of approximately 50 ng/ µ genomic".

*Response:*

*Correction has been done.*

Comment:

Line 107-108: "The fragments were ligated with T4 DNA ligase to EcoRI and MseI adapters at 37 °C for 3 h followed by 4°C overnight. ". Check the information regarding the overnight incubation temperature. Is it 4 or 16?

*Response:*

*The overnight temperature was 4°C and confirmed*

Comment:

Line 109-110: "Primers used are complementary to the adapters and restriction site with three more selective bases during pre-amplification stage". Please check this statement. The information does not match with the sequences of oligonucleotide adaptors and primers used for AFLP (Table S2).

*Response:*

*The correction has been done though lines 112 to 126*

Comment:

Line 112: "... different combinations of IR-700 labeled EcoRI primer and IR-800 MseI primer."
Of the two primers combination (EcoRI/MseI), only one labeled primer is used in the reaction, the other one is not labeled. Thus, the sentence should be rewriten to avoid misinterpretation. I suggest to include on Table 1, for each selective primer combination, which one was labeled, i.e., E-CT (IR700)/M-CAT.

*Response:*

*Correction has been made in Table 1*

Comment:

Line 145: correct the name POWERCORE

*Response:*

*Correction has been done.*

Comment:

Line 166: Check the initial number of selective primer combinations. (13 or 9?)

*Response:*

*The number of primers initially were 13. Among the evaluated primers, five primer combinations (Table 1) were used*

Comment:

Line 225: Between or among populations? Is the 6.32% value an average among the populations?

*Response:*

*About 6.32% of variability was between families. Correction has been done*
